# Transcatheter closure of a rare congenital left ventricle to right atrium shunt using the amplatzer duct occluder II: A case report

**DOI:** 10.1097/MD.0000000000022576

**Published:** 2020-11-20

**Authors:** Xiaoqing Shi, Kaiyang Wang, Jinhui Li, Jinlin Wu, Kaiyu Zhou, Yimin Hua, Yifei Li

**Affiliations:** a Key Laboratory of Birth Defects and Related Diseases of Women and Children of MOE, Department of Pediatrics, West China Second University Hospital; b State Key Laboratory of Oral Diseases, National Clinical Research Center for Oral Diseases, West China Hospital of Stomatology, Sichuan University, Chengdu, Sichuan, China.

**Keywords:** amplatzer duct occluder II, left ventricle-right atrium shunt, transcatheter closure, ventricular septal defect

## Abstract

**Rationale:**

Left ventricle-right atrium (LV-RA) shunt is a rare case and surgical repair has been the routine procedure to correct this defect. With the rapid development of transcatheter technology, some of the cases can be closed with transcatheter technique. Here, we would like to report a congenital LV-RA case who received transcatheter closure using the Amplazter duct occluder II (ADO II) and the short-term outcomes of this procedure.

**Patient concerns:**

A 2-year-old female presented a systolic murmur between the 2^nd^ to 3^rd^ sternal rib during the routine examination before kindergarten administration.

**Diagnosis:**

The patient denied any cardiac surgery, endocarditis, trauma or cardiomyopathy. The echocardiography confirmed an abnormal shunt between left ventricle and right atrium located in the superior part of ventricular septum which is closed to septal tricuspid valve and anterior mitral valve, and the diameter of this shunt is 2 mm. Besides, the dilation of right atrium (40 mm) has been identified which was not a common clinical manifestation of typical ventricular septal defect. Angiography demonstrated the shunt driven from left ventricle to right atrium.

**Intervention:**

An AGA ADO II device had been delivered to close the defect.

**Outcome:**

Follow-up kept for 3 months. Echocardiography revealed completed closure of the shunt with normal movement and function valves. And no complication of arrhythmia has been recorded.

**Lessons:**

This case report highlighted the administration of ADO II in some ventricular septal defect with superior location, and provided an essential experience of using ADO II to close long tunnel type LV-RA shunt.

## 1 Introduction

Left ventricle-right atrium (LV-RA) shunt is a rare type of case that is also called a Gerbode defect.^[1]^ LV-RA shunt has been classified as a special type of ventricular septal defect.^[2]^ Communication between the left ventricle and right atrium is the significant symptom in echocardiography examination. The defect is always limited in size and its pathophysiological and hemodynamic is mainly similar to typical ventricular septal defects. According to the reported cases, there are 2 types of LV-RA shunt: congenital and acquired.^[3,4]^ Acquired LV-RA shunt cases are always secondary to iatrogenic, infective, traumatic and ischemic causes.^[5]^ Surgical repair has been the routine procedure to correct this defect. With the rapid development of transcatheter technology, some of the cases can be closed with transcatheter technique.^[2,6]^ A review summarized that a Amplatzer duct occluder was the most common choice for such patients, while ventricular septal defect and atrial septal defect occluders have also been applied to several acquired cases. Here, we would like to report a congenital LV-RA case who received transcatheter closure using the AMPLATZERTM Duct Occluder II (ADO II) and her short-term outcomes. The written informed consent has been obtained from the patient's parents for describing the case details and demonstrating related radiological or echocardiographic images scientifically.

## 2 Case presentation

**Figure 1:**
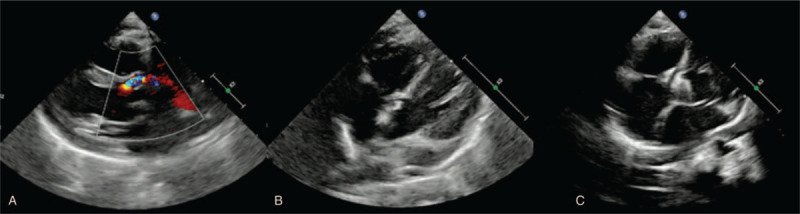
Echocardiography before and after closure of LV-RA shunt. (A) For the blood flow from left ventricle shunt into right atrium. (B) Shows a narrow tunnel for the defect. (C) Demonstrates the position of Amplatzer duct occluder II after the closure. LV-RA = left ventricle-right atrium.

**Figure 2:**
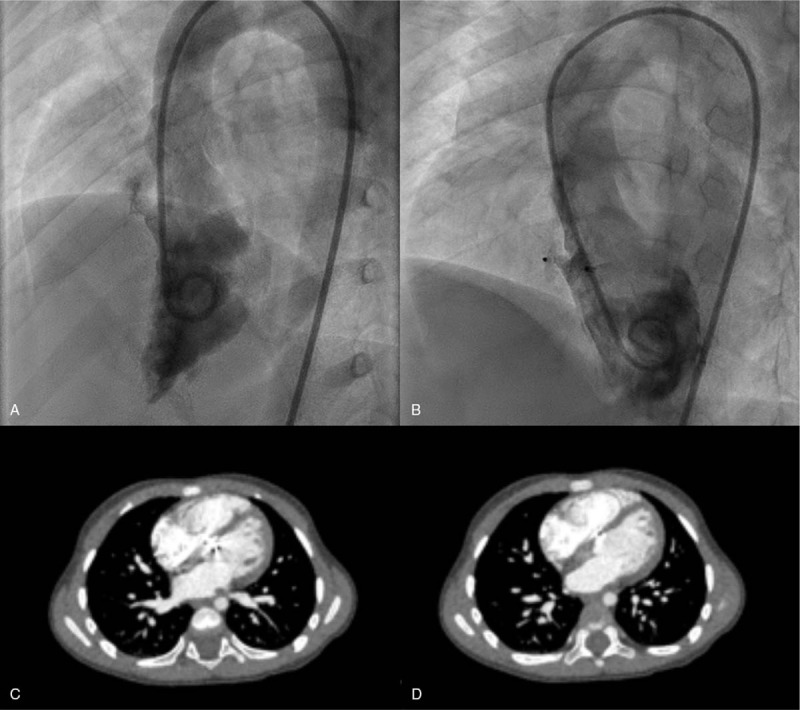
Angiography for during the procedure of LV-RA shunt closure. (A) For the angiography in left ventricle and the right atrium appears at first. (B) For the angiography after closure shows the completed closure of the defect. The CT scanning for the device position. (C and D) Show the different level of heart section. CT = computed tomography, LV-RA = left ventricle-right atrium.

A 2-year-old female presented a systolic murmur between the 2nd and 3rd sternal ribs during a routine examination before kindergarten admission. This child was referred to a cardiologist at our institute. The patient had a normal growth in height and weight and was not known to have any cyanotic, recurrent respiratory infection or heart failure symptoms. In addition, there was no past medical history of cardiac surgery, endocarditis, trauma, or cardiomyopathy. The echocardiography confirmed an abnormal shunt between the left ventricle and right atrium located in the very superior part of the ventricular septum close to the septal tricuspid valve and anterior mitral valve, with a diameter of 2 mm. Electrocardiography and Holter monitoring had been performed to demonstrate that there was no arrhythmia before the procedure. In addition, dilation of the right atrium (40 mm) was identified, which was not a common clinical manifestation of typical ventricular septal defect (Fig. 1A and B). Therefore, a rare congenital LV-RA shunt type I was suspected. In consideration of the small size of the LV-RA shunt and some previous case reports indicating that transcatheter closure would be an alternative to surgery, an invasive procedure was scheduled. During the procedure, angiography demonstrated that the right atrium first appeared with contrast medium, which then flowed into the right ventricle and pulmonary circulation (Fig. 2A). As a long ductus from the left ventricle to the right atrium was suspected, the ADO II was chosen for this case. The ADO II device is a type of self-expanding nitinol mesh device for the occlusion of patent ductus arteriosus. Its most special feature is that the ADO II device presents a great flexibility, which can result in little harm to valves and has been applied to some superior location ventricular septal defect closures in consideration of the interaction with aortic valves. Finally, an AGA ADO II 3/4 mm device was delivered from the 4F sheath, and the defect was closed successfully (Fig. 2B). One week after the procedure, echocardiography revealed completed closure of the shunt with normal movement and function of tricuspid valves, mitral valves, and aortic valves (Fig. 1C). One month after that, enhanced computed tomography scanning confirmed that the occluder was in the appropriate position (Fig. 2 C and D). Echocardiography was repeated at 3 months post closure and revealed normal device position, heart structure, and function. In addition, as arrhythmia can occur after transcatheter closure, ECG was performed at 1 week, 2 weeks, 4 weeks, and 3 months post closure, while an additional Holter monitoring series was completed at 4 weeks, which indicated that there was no arrhythmia occurrence. According to our follow-up data, there is no impairment of tricuspid valvar movement due to the implantation of the ADO II device. Thus, the highlight of this technique is that it allows the patient to receive an transcatheter cardiac interventional closure and maintain normal valvar function, avoiding open heart surgery.

## 3 Discussion and conclusion

Identification of congenital LV-RA shunt is rare. However, due to its special location, the transcatheter closure device can occupy the space and allow for the normal movement of tricuspid valves, mitral valves, and aortic valves using the typical AGA Amplatzer ventricular septal defect occluder. Thus, for most cases, even for acquired LV-RA shunt, received surgical repair.^[1]^ With the approval of the ADO II occluder, it has become an alternative for ventricular septal defect closure, which need less space and did not alter the movement of surrounding valves. In our institute, the ADO II occluder has been applied in the closure of subarterial ventricular septal defects whose sizes are smaller than 4 mm, small perimembranous ventricular septal defects with superior position and long tunnel defects. In the middle- and long-term follow-up, the ADO II occluder demonstrated a minimal impact on the valves, and no residual shunt was confirmed. Among the most dangerous complications of transcatheter closure ventricular septal defect, atrioventricular block has not been recorded,^[7,8]^ and only some cases presented incomplete right branch bundle blocks. Therefore, it is believed that the ADO II would be a smart option for ventricular septal defect closure.

For this patient, we excluded all the possible reasons that could induce a required LV-RA shunt and ultimately reached a diagnosis of congenital shunt. One review summarized congenital LV-RA cases.^[5]^ And among the reported population, the youngest reported case of congenital LV-RA shunt had been received surgical repair at age around 2 years, similar to the current patient. As the right atrium dilation was identified by echocardiography, we considered that closure timing would be acceptable.

Based on the anatomical characteristics of congenital LV-RA shunt, the defect could be quite close to the tricuspid valves. Any larger, sharp and solid materials could cause damage and influence valvar movements.^[9]^ Additionally, the Amplatzer duct occluder and ventricular septal defect occluder are made with very solid fibers, which might cause mechanical injuries to the valves and encroach upon the space for their opening and closure; thus, these occluders were abandoned for this patient. However, the ADO II occluder was applied in this patient and has achieved promising outcomes both immediately and during the short-term follow-up. Herein, we presented the following indications for the application of the ADO II for LV-RA shunt:

(1) type I LV-RA shunt^[2]^ has been confirmed in both echocardiography and angiography images,
(2) the small-size defect was less than 3 mm, and
(3) there were no severe impairments in tricuspid or aortic valve movement.

This technique is the same as the procedure for ventricular septal defect transcatheter closure. So that, the surgeons who can perform ADO II closure of LV-RA shunt, need complete the training for typical ventricular septal defect closure.

The congenital LV-RA shunt is a rare defect; affected patients might be asymptomatic, with a similar hemodynamic change as those with small perimembranous ventricular septal defects. During echocardiography and angiography examinations, careful observation should be applied to arrive at the correct diagnosis. The ADO II could be an acceptable option to close this defect. This approach might provide safety and efficacy benefits in reducing valve-related complications in ventricular septal defect closure during follow-up.

## Author contributions

**Conceptualization:** Xiaoqing Shi, Yifei Li.

**Data curation:** Xiaoqing Shi.

**Formal analysis:** Xiaoqing Shi, Jinhui Li, Yifei Li.

**Funding acquisition:** Yifei Li.

**Investigation:** Xiaoqing Shi, Kaiyang Wang, Jinhui Li.

**Methodology:** Kaiyang Wang.

**Project administration:** Jinlin Wu, Yifei Li.

**Resources:** Kaiyu Zhou.

**Supervision:** Jinlin Wu, Yimin Hua.

**Validation:** Kaiyu Zhou.

**Writing – original draft:** Yifei Li.

**Writing – review & editing:** Yifei Li.
